# Heat and salinity stress on the African eggplant F1 Djamba, a Kumba cultivar

**DOI:** 10.3389/fpls.2024.1323665

**Published:** 2024-02-14

**Authors:** Noémie David-Rogeat, Martin R. Broadley, Eleftheria Stavridou

**Affiliations:** ^1^ Department of Plant Sciences, School of Biosciences, University of Nottingham, Nottingham, United Kingdom; ^2^ Crop Science and Production Systems, NIAB, Kent, United Kingdom; ^3^ Rothamsted Research, Harpenden, United Kingdom

**Keywords:** *Solanum aethiopicum*, salinity, heat-wave, stomatal conductance, antioxidants

## Abstract

Climate change is expected to increase soil salinity and heat-wave intensity, duration, and frequency. These stresses, often present in combination, threaten food security as most common crops do not tolerate them. The African eggplant (*Solanum aethiopicum* L.) is a nutritious traditional crop found in sub-Saharan Africa and adapted to local environments. Its wider use is, however, hindered by the lack of research on its tolerance. This project aimed to describe the effects of salinity (100 mM NaCl solution) combined with elevated temperatures (27/21°C, 37/31°C, and 42/36°C). High temperatures reduced leaf biomass while cell membrane stability was reduced by salinity. Chlorophyll levels were boosted by salinity only at the start of the stress with only the different temperatures significantly impacted the levels at the end of the experiment. Other fluorescence parameters such as maximum quantum yield and non-photochemical quenching were only affected by the temperature change. Total antioxidants were unchanged by either stress despite a decrease of phenols at the highest temperature. Leaf sodium concentration was highly increased by salinity but phosphorus and calcium were unchanged by this stress. These findings shed new light on the tolerance mechanisms of the African eggplant under salinity and heat. Further research on later developmental stages is needed to understand its potential in the field in areas affected by these abiotic stresses.

## 1 Introduction

The last decade has been marked by increasing temperatures and longer, more intense, and more frequent heatwaves around the world, in particular in Africa (Pörtner et al., 2022). High temperatures threaten food security as most crops grown worldwide are heat-sensitive (Hassan et al., 2021). The yield reduction is due to various mechanisms triggered by heat including the reduction of leaf expansion and gas exchange to limit water loss alongside the damages to enzymes and other key parts of the photosynthesis system due to the high leaf temperature (Hassan et al., 2021). In addition, soil salinization is increasing worldwide due to a combination of natural and man-made processes (Pörtner et al., 2022). Soil salinity also negatively impacts crop growth, leading to stunted growth and poor yields due to limited water uptake and ion imbalance (Ondrasek et al., 2022).

Under field conditions, heat and salinity often occur concurrently, especially in coastal areas. While soil salinity leads to an increase in sodium ion uptake, limiting the uptake of essential nutrients and toxic for cells, heat destroys cell membranes through the denaturation of enzymes and other compounds. These two highly damaging processes have antagonistic activity due to the key role of membranes in nutrient uptake and translocation within the plant, leading to a negative impact of the stress combination (Nadeem et al., 2022). The interaction of most processes under the combination of heat and salinity is still largely unknown. For example, heat stress might promote stomatal conductance to regulate leaf temperature but salinity stress tends to decrease it to limit water loss. It is therefore important to study their combination to understand the mechanisms in place and be able to accurately predict the impact of environmental stress on crop growth.

The introduction of tolerant and diverse crops in food production systems is crucial to enhancing food security and consumers’ nutrition. Indigenous vegetables are generally more tolerant to stress than their exotic counterparts due to their selection in harsh environments and their large genetic assortment (Akinola et al., 2020). For example, wild tomatoes *Solanum cheesmaniae* L. Ridley displayed higher salinity tolerance than the commercial tomato by maintaining leaf water content, improving leaf elongation and potassium uptake, and limiting sodium ions uptake (Pailles et al., 2020). Even though indigenous vegetables have been poorly studied in the past, they have strong local importance and include all types of crops including legumes, cereals, and vegetables (Akinola et al., 2020). *Solanum aethiopicum* L., the African eggplant, is indigenous to Africa and present in multiple forms across sub-Saharan Africa, displaying a great genetic diversity and nutrition (Han et al., 2021). It is liked for its nutritional quality and is an important income stream for small-scale farmers across the African continent (Han et al., 2021). The African eggplant has been selected by the World Vegetable Center as a priority crop for breeding strategies to improve field resilience and diversity (Dinssa et al., 2016). The African eggplant’s salinity tolerance was low, however, as reported by de Fatima et al. (2023) with reductions in growth and gas exchange of more than 10% observed when soil salinity was above 1.37 dS m^-1^. Interestingly, stomatal conductance was enhanced by salinity, an increase explained by the energy re-deployment from plant growth to stomatal dynamics (de Fatima et al., 2023). In contrast, a decrease in stomatal conductance and photosynthetic parameters were noted in another African eggplant cultivar under heat (Nkansah, 2001). The vegetative growth, in this case, increased between 30°C and 40°C, suggesting contrasting impacts of heat and salinity in the African eggplant (Nkansah, 2001).

New varieties of the African eggplant are being developed industrially but lack research on their abiotic stress tolerance to ensure their relevance in the field in stress-prone areas. This is the case of the cultivar F1 Djamba developed by Novagenetic (Technisem, Longué-Jumelles, France) and promoted for its high yield. Technisem market includes tropical areas in Northern and Western Africa, which are highly likely to experience a high heat increase due to climate change alongside a constant increase in soil salinity (World Meteorological Organization, 2022). Understanding how F1 Djamba responds to the combination of stresses is thus crucial to ensure its use in every possible area. In addition, its response will be useful to get a better understanding of how the African eggplant responds to stress for future breeding.

This study aimed to understand how soil salinity and heat affect the African eggplant cv. F1 Djamba when present individually and in combination. Leaf biomass, photosynthesis parameters, and leaf biochemical analysis including proteins, antioxidants, and nutrient concentrations were assessed to clarify some stress-related mechanisms used by the African eggplant. It was hypothesized that the combination of heat and salinity would lead to specific responses not observed when the stresses were present individually. This research will be useful to understand how this plant can be used in future climates.

## 2 Materials and methods

### 2.1 Plant material and growth conditions

The combination of salinity stress and high temperatures on the African eggplant Kumba (*Solanum aethiopicum* L. cv., F1 Djamba) was investigated. F1 Djamba seeds, generously provided by Novagenetic (Technisem, Longué-Jumelles, France), were sown in modular trays in John Innes No.2 soil compost (7 loam:3 peat:2 sand) (Westland^®^, J Arthur Bower’s) and placed in an MLR-352 growth cabinet (PHC Holdings Corporation^®^, Tokyo, Japan). The environmental conditions were set at 70% relative humidity, 27/21°C for a 12 h photoperiod. After four weeks, 24 homogenous seedlings were transplanted in 3 L pots (at a rate of one plant per pot) in John Innes No.2 soil and placed following a split-plot design in three growth cabinets (Plant growth chamber A1000, Conviron^®^, Winnipeg, Canada) fitted with Valoya LEDs lights (BX NSI spectrum ‘white’ LED, Valoya^®^, Helsinki, Finland) providing 350 *µ*mol m^-2^ s^-1^ light intensity. The growing conditions stayed the same.

### 2.2 Treatment application

**Figure 1:**
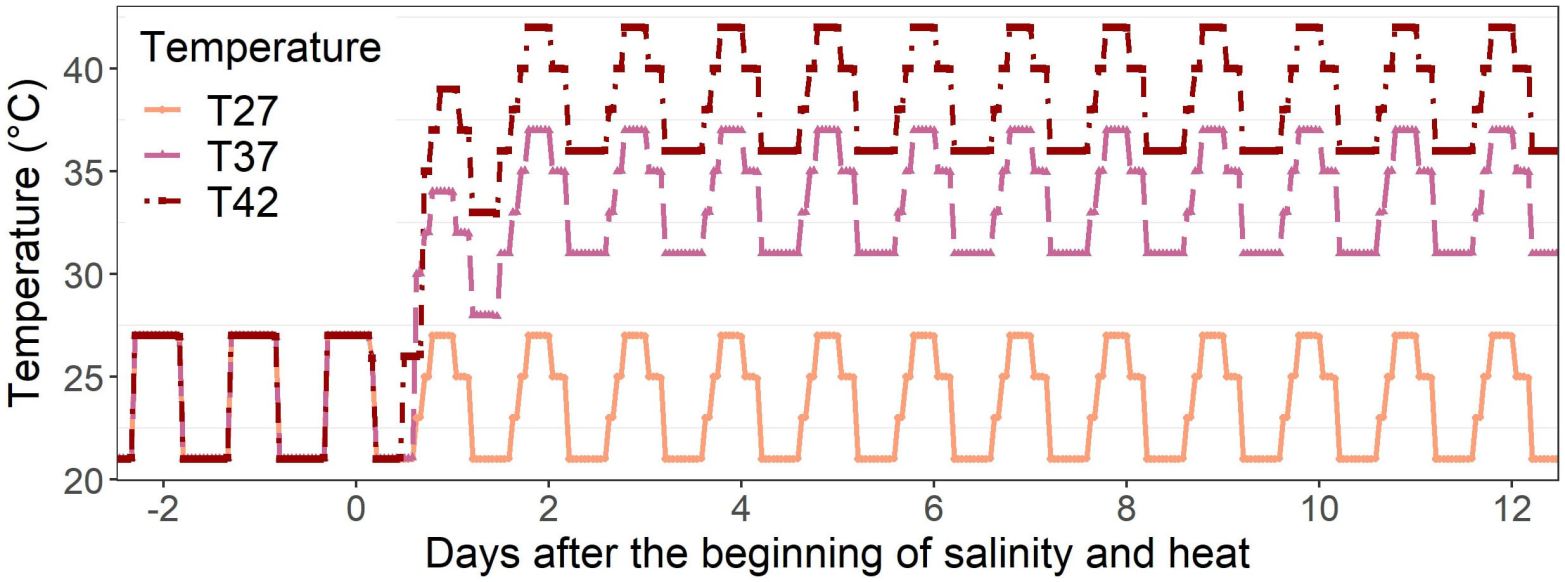
Daily temperature schedule of 27°C (T27), 37°C (T37), and 42°C (T42) treatments throughout the experimental period (from -2 DASH to 12 DASH).

After 11 days of acclimation under optimal conditions, salinity stress was applied to four plants per cabinet by watering them using 100 mM NaCl solution while the other four plants, used as the control, received only water. This sodium chloride concentration corresponds to a soil electrical conductivity of around 9.8 dS m^-1^, considered highly saline (West, 1975). As the stress was applied as irrigation for only 12 days, rather than an initial mix of the salt into the soil, this high level was selected to ensure a significant increase in soil salinity. All the plants were irrigated with the same amount of NaCl. At the same time, considered to be 1 DASH (days after salinity and heat), heat stress was applied in two of the cabinets with a maximum of 37/31°C (T37) and 42/36°C (T42) day/night temperature when compared to 27/21°C (T27) for the control plants, a treatment also maintained for 12 days. These increased temperatures were selected based on previous research on the African eggplant (data not shown) and on temperatures sometimes reached in Northern and Western Africa during heat waves, with T42 also being higher than the average monthly temperature of most countries in Northern and Western Africa to mimic extreme conditions (Worlddata, 2023). To ensure plants were well-watered throughout the experiment, soil water content was monitored using an HH2 moisture meter attached to a WET sensor (Delta-T devices^®^, Burwell, UK). The daily schedules of each treatment are shown in **Figure 1**. The experiment was repeated once with four replicates per treatment each time.

### 2.3 Growth and physiological measurements

#### 2.3.1 Leaf expansion rate

Three times a week, the length and maximum width of two labelled leaves were recorded. These leaves were originally labelled at a similar height and developmental stage and were less than 30 mm in length. The length and width leaf expansion rate (LER) were considered to be the growth difference between the repeated measurements.

#### 2.3.2 Chlorophyll fluorescence and gas exchange

Leaf fluorescence parameter SPAD, measuring the transmittance of red and infrared through the leaf, and photosynthetic parameters photosystem II quantum yield (*ϕ*2) and quantum yields of non-photochemical exciton quenching (*ϕ*NPQ) were measured three times a week 5 h after the start of the photoperiod from 0 to 12 DASH using a MultispeQ V2.0 device with Photosynthesis RIDES 2.0 protocol (PhotosynQ^®^, East Lansing, MI, United States) (Kuhlgert et al., 2016). Leaf chlorophyll, flavonoids, and nitrogen balance were measured three times a week at the same time from 0 to 12 DASH using a Dualex^®^ device (ForceA, Montpellier, France). Stomatal conductance was recorded at 4 h after the start of the photoperiod at 0, 1, 7 and 12 DASH using a leaf porometer (Model SC-1, Decagon Devices, METER group, Pullman, WA, United States). At 12 DASH, diurnal measurements were taken at 0, 4, 8, and 12 h after the start of the photoperiod.

#### 2.3.3 Destructive harvest

On the last day of the experiment, 12 DASH, a leaf sub-sample representing a mix of developing and developed leaves was removed from the plant, placed in liquid nitrogen straight after recording its combined weight, and stored at -80°C for biochemical analysis. Leaves and stems were weighed separately, after measuring leaf number, stem diameter and plant height, and oven-dried at 80°C until reaching a constant weight.

#### 2.3.4 Leaf electrolyte leakage

One leaf disc (10 mm diameter) was cut out from five fully expanded leaves on each plant at the end of the treatment (12 DASH). The discs were washed three times with distilled water to remove surface contamination and placed in 20 mL of distilled water. The discs were incubated at room temperature on a shaker at 200 rpm for 24 h and the electrical conductivity of the bathing solution (EC1) was eventually recorded using a LAQUAtwin EC-33 meter (Horiba^®^, Kyoto, Japan). The discs were then placed in a water bath at 95°C for 60 min and a second electrical conductivity reading (EC2) was taken after cooling the solution to room temperature. The electrolyte leakage (EL), expressed as % EL, was calculated as per **Equation 1**:


(1) EL=EC1/EC2∗100

### 2.4 Soil electrical conductivity

On the last day of the experiment, a soil sample from each pot was extracted and left to air-dry until reaching constant weight. Five grams of the dry soil was then added to 25 mL of distilled water, shaken, and left to settle for 24 h as described in He et al. (2012). The electrical conductivity of the supernatant (EC 1:5) was then measured using a LAQUAtwin EC-33 meter (Horiba^®^).

### 2.5 Biochemical analysis

#### 2.5.1 Leaf chlorophyll content

Leaf chlorophyll content was determined following the method described in Wintermans and De Mots (1965). At 12 DASH, two leaf discs (1.54 cm^2^ each) from the first two fully expanded leaves were immersed in 10 mL 95% cold ethanol (Fischer Scientific, Hampton, NH, United States) and kept at 4°C in the dark for 48 h. The absorbance of the supernatant was read at 470, 649, and 665 nm using a UV-VIS spectrophotometer (Ultrospec III, Pharmacia LKB, Stockholm, Sweden). The amount of chlorophyll a (Chl a) and b (Chl b) and carotenoids (Car) per unit area were calculated following **Equations 2**–**4**:


(2) Chl a= (13.95∗A649)/1.54

(3) Chl b=(24.96∗A649−7.32∗A665)/1.54

(4) $$Car=\;(10^{3}*A470-2.05*Chl\;a-114.8*Chl\;b)/1.54$$

#### 2.5.2 Total carbohydrates

The amount of soluble sugars in plants at 12 DASH was quantified using the phenol-sulfuric acid colorimetric method (Dubois et al., 1956). A dry leaf sample (50 mg) was dissolved in 5 mL of 80% cold methanol (Fischer Scientific). Subsequently, 100 *µ*L of the resulting solution was combined with 900 *µ*L of distilled water, 2.5 mL of sulfuric acid (Fischer Scientific), and 500 *µ*L of a 5% phenol (Fischer Scientific) solution. After incubating for 20 min at room temperature, the absorbance was measured at 490 nm using a UV-VIS spectrophotometer (Ultrospec III). Glucose (Merck, Darmstadt, Germany) served as the standard.

#### 2.5.3 Total antioxidants

The TEAC (Trolox Equivalent Antioxidant Capacity) method was used to determine total antioxidants (Re et al., 1999). A solution of 7 mM ABTS (Merck) with 2.45 mM potassium persulfate (Merck) was made and left in darkness overnight. This solution was then diluted with ethanol (Fischer Scientific) until reaching an absorbance of 0.9 at 734 nm and warmed on a hotplate at 40°C. Fifty milligrams of dry leaf sample (extracted at 12 DASH) was diluted in 5 mL of 80% cold methanol (Fischer Scientific) and 30 *µ*L of the sample supernatant was pipetted in 3 mL of the diluted working solution. After 15 min in a 40°C water bath, the absorbance of the samples was read at 734 nm in a UV-VIS spectrophotometer (Ultrospec III).

#### 2.5.4 Total phenols

The Folin-Ciocalteau method described by Singleton et al. (1999) was used to measure leaf phenol content on the final day of the experiment (12 DASH). Cold methanol (80%, Fischer Scientific) was used to digest 50 mg of dry leaf sample and 300 *µ*L of the digested sample was added to 600 *µ*L of 10% Folin-Ciocalteau reagent (Merck) and left to stand for 2 min. Then, 2.5 mL of 700 mM sodium carbonate (Fischer Scientific) was added. The mixture was left to stand at room temperature for 1 h, after which the absorbance of each sample was read at 765 nm in a UV-VIS spectrophotometer (Ultrospec III). Gallic acid (Merck) was used as a standard.

#### 2.5.5 Leaf nutrient concentration

Dried and milled leaf sample (300 mg), extracted at 12 DASH, was digested in 6 mL of nitric acid and placed in a digestion microwave (Multiwave PRO, Anton Paar, Graz, Austria) reaching 175°C over a 20 min period and maintained for a further 20 min followed by a 10 min cool down at 55°C. This digested extract was diluted by a factor of 36 by adding 18.2 milliQ water for multi-element analysis by ICP-MS (Inductively Couple Plasma Mass Spectrophotometry, Thermo-Fisher Scientific iCAP-Q; Thermo Fisher Scientific, Germany). Calibration standards included a multi-element solution (Claritas-PPT grade CLMS-2 from SPEX Certiprep Inc., Metuchen, NJ, United States) and a bespoke external multi-element calibration solution (PlasmaCAL, SCP Science, Quebec, Canada).

A FlashEA^®^1112 elemental analyzer (Thermo Fischer Scientific, Waltham, MA, United States) was used to determine leaf nitrogen concentration by burning 50 mg of dried and milled samples placed in a foil capsule. The gas mixture generated by combustion was filtrated and elemental nitrogen was detected by conversion, providing a nitrogen percentage.

### 2.6 Statistical analysis

Statistical studies were performed on R version 4.1.2. The data was assessed for normality and variance homogeneity of residuals. Normality was checked using a combination of the Shapiro test and visual evaluation (density plot, Q-Q plot, and histogram). Variance homogeneity was assessed using Levene’s test and visual inspection of the residuals. When the requirements were met, a two-way analysis of variance (ANOVA) test was performed on continuous data using “Salinity” and “Temperature” as the main factors and including their interaction. Tukey adjustment at a 0.05 significance level was used to test for pairwise comparison among and across groups. Whenever the interaction of stresses was significant, the interaction parameter was used for posthoc tests and letters were used to show the significance throughout the manuscript for clarity. A linear mixed-effects model was also used to analyze time series of continuous data including the interaction of the parameters of interest with time and the repetitive measurement of each plant as a random factor. For count data, a generalized linear model fitted with a Poisson distribution was checked for overdispersion and used for analysis with “Salinity” and “Temperature” as interacting factors.

The log values of leaf sodium concentration were used for statistical analysis to improve residuals’ normality and homogeneity due to the extreme treatment effect observed on the original dataset.

## 3 Results

**Figure 2:**
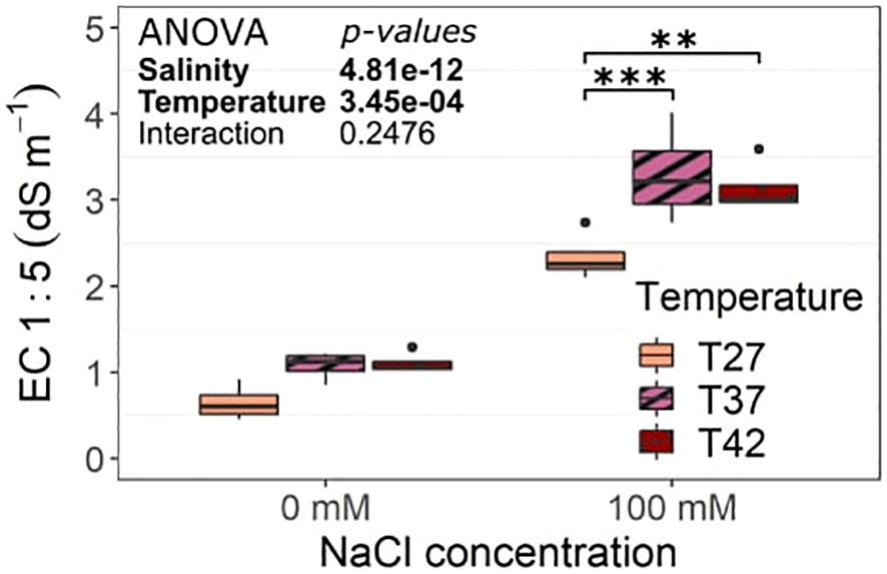
Soil electrical conductivity (EC) 1:5 at the end of the salinity treatment. The data are represented by the lower and upper whiskers, which extend to a maximum of 1.5 x Interquartile range, 25% and 75% quartiles, and median (n = 4). Stars denote the significance level based on the Tukey pairwise comparison at 95% confidence level with **p<.01 and ***p<.001. Non-significant interactions are not shown for clarity.

The salinity treatment significantly increased soil EC 1:5 at every temperature (**Figure 2**). In addition, T37 and T42 had a significantly higher soil EC 1:5 than T27 under salinity (**Figure 2**).

### 3.1 Plant development

**Figure 3:**
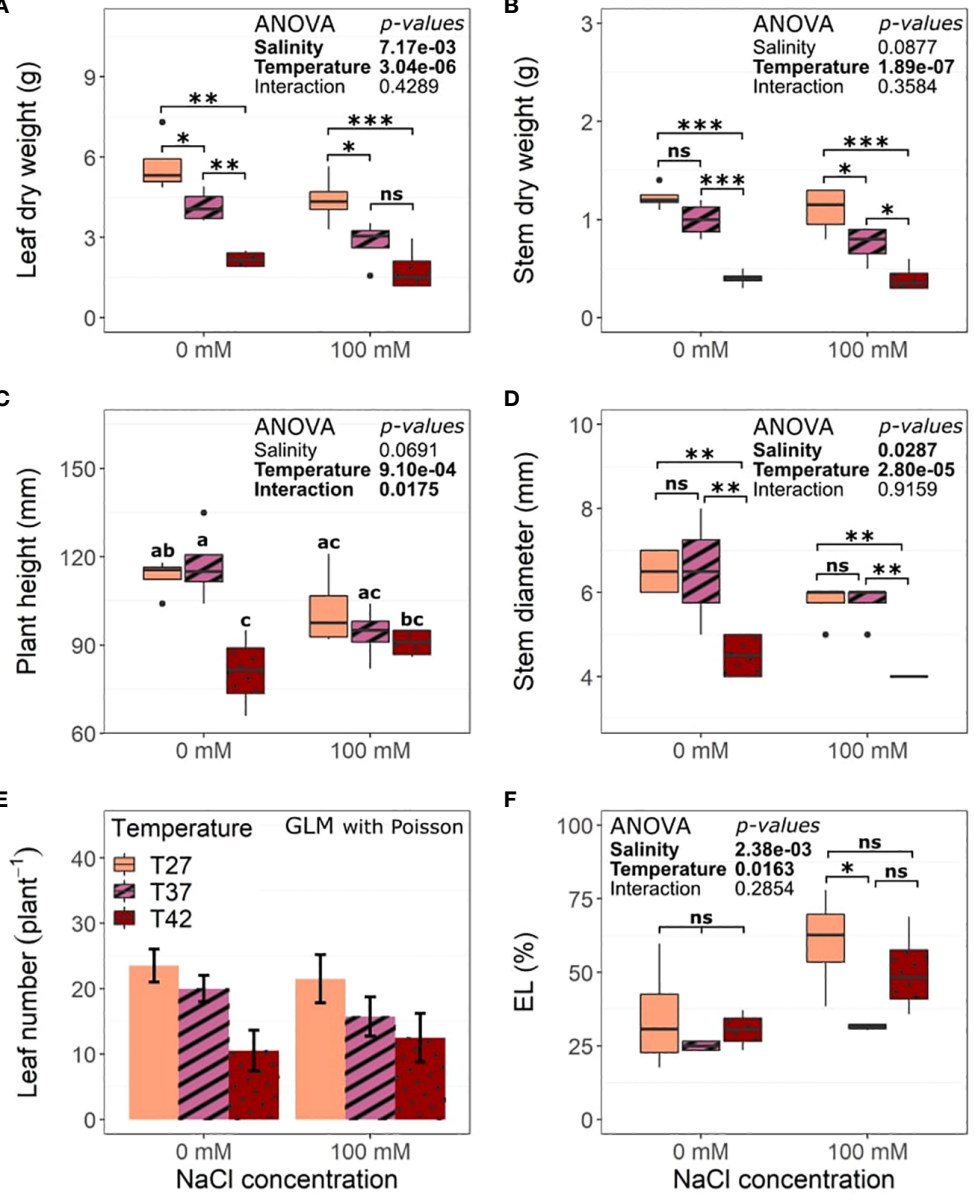
Morphological characteristics of the African eggplant at the end of the 12 days under salinity and increased temperature. Sub-plots represent **(A)** leaf dry weight, **(B)** stem dry weight, **(C)** plant height, **(D)** stem diameter, **(E)** number of leaves per plant and **(F)** electrolyte leakage (EL). The continuous datasets are represented by the lower and upper whiskers, which extend to a maximum of 1.5 x Interquartile range, 25% and 75% quartiles, and median (n = 4). Black dots represent outliers. Stars denote the significance level based on the Tukey test at 95% confidence level with * for p<.05, **p<.01, and ***p<.001. For measures where the interaction of the parameters was significant after the ANOVA, letters are used to display the difference between treatments for clarity. Boxplots not sharing any letters are significantly different in these cases. Count data are represented as the mean +/- standard deviation. ns, Non significant.

A linear decrease of leaf dry weight under increasing temperature was observed under both saline and non-saline conditions and salinity also decreased it (**Figure 3A**). Stem dry weight was maintained at T37 and reduced at T42 under 0 mM NaCl but was reduced for both T37 and T42 under 100 mM NaCl when compared to T27 (**Figure 3B**). Salinity did not impact stem dry weight. Plant height was only reduced by T42 under 0 mM NaCl conditions while plants under salinity were not impacted when compared to the control (**Figure 3C**). Salinity also impacted shoot development by reducing stem diameter while only the highest temperature reduced it in both 0 and 100 mM NaCl treatments (**Figure 3D**). Leaf number followed a similar pattern and was only reduced by T42 under both 0 and 100 mM NaCl conditions (**Figure 3E**).

Electrolyte leakage was increased by salinity but not significantly impacted by the temperatures at 0 mM NaCl and reduced by T37 when compared to T27 under 100 mM NaCl (**Figure 3F**).

**Figure 4:**
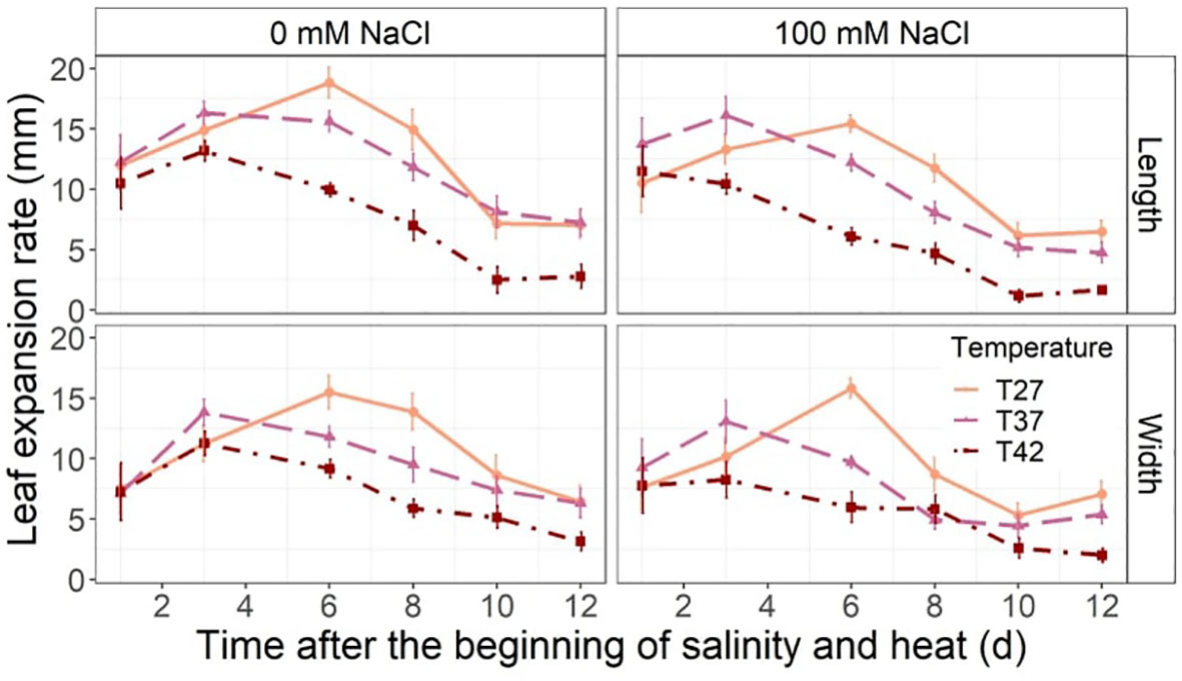
Leaf length (top row) and width (bottom row) expansion rate throughout the salinity and increased temperature. The data are shown as mean +/- standard error.

While an increase in both length and width LER was observed from the 1^st^ day of the treatment to the 3^rd^, only T27 plants continued to increase up to day 6 with T37 and T42 growth rates reducing slightly (**Figure 4**). From the 6^th^ day, T42 treatment reduced length LER significantly when compared to T27 in both salinity treatments but only reduced width LER of plants under 0 mM NaCl on day 6 and 8 (**Figure 4**). Salinity reduced length LER on day 6 at every temperature (**Figure 4**). On day 8, length and width LER were only reduced for T27 and T37 plants by salinity and no further impact was noted for the rest of the experiment due to salinity (**Figure 4**). Both plants under saline and non-saline conditions had the same pattern of initial LER increase before slowing down and stabilizing.

### 3.2 Leaf chlorophyll and polyphenols

**Figure 5:**
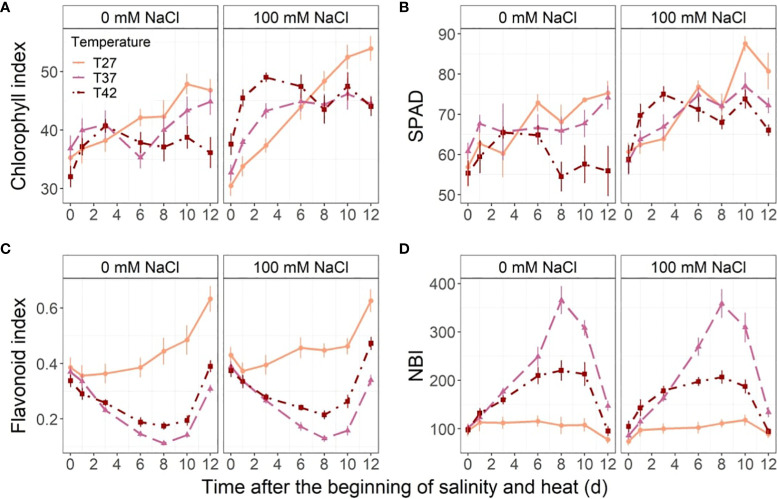
Leaf chlorophyll and polyphenols throughout the stress period. Sub-plots represent **(A)** chlorophyll index, **(B)** SPAD, **(C)** flavonoid index and **(D)** nitrogen balance index (NBI). The data are shown as mean +/- standard error.

Salinity increased chlorophyll index throughout the stress (**Figure 5A**). Under 100 mM NaCl, chlorophyll index increased more quickly under T37 and T42 than T27 until 4 DASH and stabilized thereafter. The chlorophyll index of T27 plants, however, continued to increase until the end of the experiment and surpassed T37 and T42 levels at 8 DASH (**Figure 5A**). Although at no-saline conditions the chlorophyll index was not statistically different throughout the stress period between the temperature treatments, the T42 plants had lower levels than the other two temperatures at 12 DASH (**Figure 5A**). Salinity increased SPAD index halfway through the experiment with significant differences appearing from day 6 until day 10 (**Figure 5B**). The temperature stress also affected SPAD under 0 mM NaCl, with a decrease from day 8 on plants grown at T42 (**Figure 5B**). Under 100 mM NaCl, T42 only significantly reduced SPAD on day 10 and day 12 (**Figure 5B**). T42 reached its peak SPAD earlier than the other temperatures at 100 mM NaCl (**Figure 5B**).

Neither flavonoids nor nitrogen balance index were impacted by salinity, but they decreased and increased, respectively, under T37 and T42 from the 3^rd^ day of stress (**Figures 5C, D**). Temperature decreased flavonoids from day 3 to 8, after which the T37 and T42 levels started to increase without fully recovering (**Figure 5C**). While the nitrogen balance index at T27 remained stable throughout the experiment, both T37 and T42 increased from day 3 to day 8 (**Figure 5D**). From day 8, the nitrogen balance index of T37 and T42 started to decrease and eventually reached the same level as T27 at the end of the experiment (**Figure 5D**). The highest nitrogen balance index was reached under the T37 treatment on day 8 while the T42 treatment effect was more moderate (**Figure 5D**).

**Figure 6:**
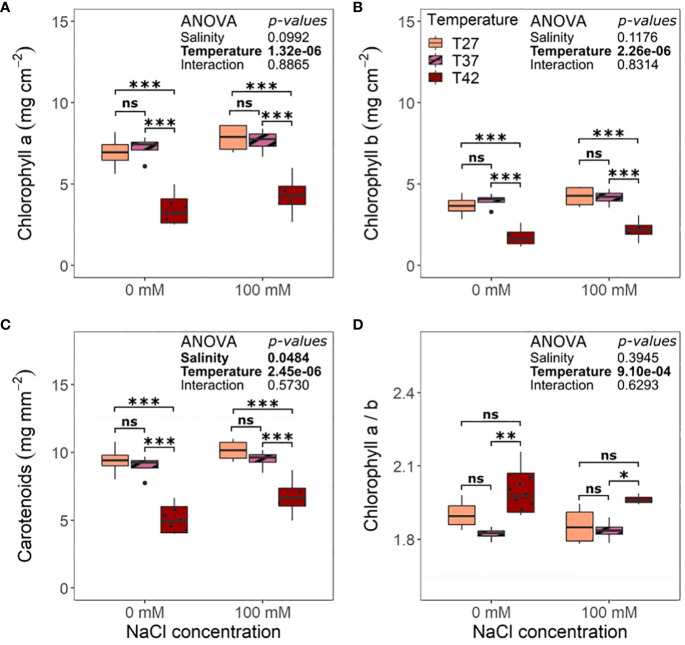
Leaf pigment content per unit area of the African eggplant after 12 days under salinity and increased temperature. Sub-plots represent **(A)** chlorophyll a, **(B)** chlorophyll b, **(C)** carotenoids, and **(D)** the ratio of chlorophyll a over chlorophyll b. The data are represented by the lower and upper whiskers, which extend to a maximum of 1.5 x Interquartile range, 25% and 75% quartiles, and median (n = 4). Black dots represent outliers. Stars denote the significance level based on the Tukey test at 95% confidence level with * for p<.05, **p<.01, and ***p<.001. ns, Non significant.

Salinity did not impact final chlorophyll content (**Figure 6**). The highest temperature, on the other hand, decreased chlorophyll a, b, and carotenoids (**Figures 6A–C**). The ratio of chlorophyll a over b was increased for T42 plants compared to T37 but not T27 (**Figure 6D**).

### 3.3 Stomatal conductance and leaf fluorescence

**Figure 7:**
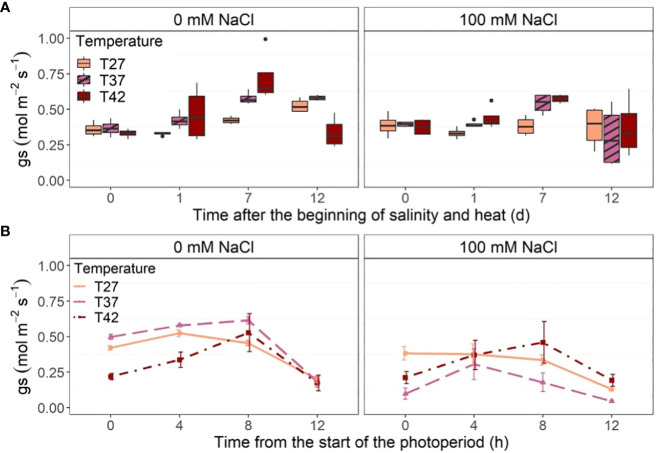
Stomatal conductance (gs) of the African eggplant. Sub-plots represent **(A)** midday stomatal conductance throughout the experiment and **(B)** diurnal stomatal conductance throughout the last day of the experiment (12 DASH). The data in **(A)** are represented by the lower and upper whiskers, which extend to a maximum of 1.5 x Interquartile range, 25% and 75% quartiles, and median (n = 4). Black dots represent outliers. The data in **(B)** are shown as mean +/- standard error.

Midday stomatal conductance increased significantly only for T37 at 100 mM NaCl when compared to the control at 1 DASH (**Figure 7A**). At 7 DASH, however, T42 at 0 and 100 mM NaCl was significantly higher than the control but not T37 (**Figure 7A**). The temperature treatment had the biggest effect on midday stomatal conductance on day 7 with both saline and non-saline T37 and T42 plants reaching their highest level then (**Figure 7A**). No differences between treatments were recorded on the last day of the experiment. Stomatal conductance of T42 plants dropped significantly on that day and stayed stable for T27 and T37 (**Figure 7A**). When looking at diurnal stomatal conductance throughout the last treatment day (12 DASH), the strongest difference was noted at the beginning of the day with a low stomatal conductance under T42 at 0 mM NaCl and under both T37 and T42 at 100 mM for the first hour of light (0 h) (**Figure 7B**). As the day progressed, stomatal conductance increased quickly in plants under the T42 treatment while plateaued, or even reduced, in the other temperatures (**Figure 7B**). The T42 treatment thus reached the same stomatal conductance as T27 and T37 eight hours after the start of the photoperiod at 0 mM NaCl (**Figure 7B**). T42 plants also reached their peak stomatal conductance eight hours after the beginning of the photoperiod at 100 mM NaCl (**Figure 7B**). At 0 mM NaCl, T37 stomatal conductance also peaked 8 h after the beginning of the photoperiod and was significantly higher than T27 (**Figure 7B**). The T27 plants reached their peak stomatal conductance earlier, at 4 hours after the beginning of the photoperiod. Salinity mostly affected T37 plants, which had the lowest stomatal conductance at every time point, even though only significant at the beginning and the end of the photoperiod (**Figure 7B**).

**Figure 8:**
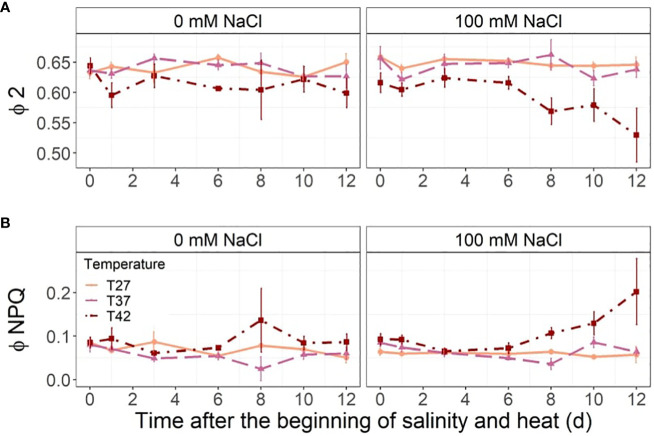
Leaf fluorescence parameters throughout the salinity and increased temperature. Sub-plots represent **(A)** quantum yield of photosystem II (*ϕ* 2) and **(B)** ratio of incoming light going through nonphotochemical quenching (*ϕ* NPQ). The data are shown as mean +/- standard error.

The T42 treatment decreased the quantum yield of photosystem II (*ϕ*2) at 1, 6, 8 and 12 DASH under both salinity and no salinity treatments (**Figure 8A**). This was exacerbated by salinity towards the end of the stress period (**Figure 8A**). The ratio of incoming light going through non-photochemical quenching (*ϕ*NPQ) was only increased by the combination of salinity and the highest temperature at the end of the experiment at 10 and 12 DASH (**Figure 8B**). The *ϕ*NPQ and *ϕ*2 remained constant throughout the stress period for T27 and T37 at both salinity levels (**Figure 8**).

### 3.4 Biochemical analysis

**Figure 9:**
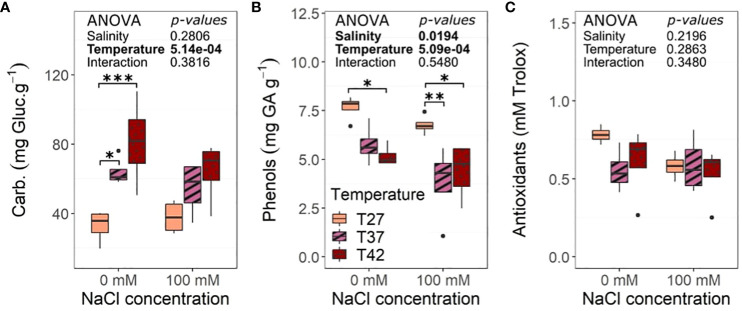
Biochemical analysis of the African eggplant dry leaves at the end of the 26 days under salinity increased temperature. Sub-plots represent **(A)** total carbohydrates, **(B)** phenols, and **(C)** total antioxidants activity. The data are represented by the lower and upper whiskers, which extend to a maximum of 1.5 x Interquartile range, 25% and 75% quartiles, and median (n = 4). Stars denote the significance level based on the Tukey test at 95% confidence level with * for p<.05, **p<.01, and ***p<.001. Non-significant interactions are not shown for clarity. Carbs, Carbohydrates; Gluc, Glucose; GA, Gallic Acid.

Total carbohydrates increased under T37 and T42 at 0 mM NaCl condition when compared to T27 plants, but that was not observed at 100 mM NaCl (**Figure 9A**). Total phenols were impacted by the temperatures with a decrease as temperature increased in both saline and non-saline treatments while salinity also decreased its levels (**Figure 9B**). The decrease in phenol content was noted for both T37 and T42 under 100 mM NaCl but was only significant for T42 under 0 mM NaCl treatment (**Figure 9B**). Total antioxidants were unchanged by either stress or their interaction (**Figure 9C**).

Leaf sodium concentration increased significantly for plants grown under 100 mM NaCl (**Table 1**). Both T37 and T42 increased the leaf sodium concentration when compared to T27 while T37 and T42 were not statistically different (**Table 1**). Leaf nitrogen concentration was reduced as temperature increased and when under salinity (**Table 1**). Leaf phosphorus concentration was unaffected by the salinity treatment but was increased by T37 and T42 when compared to T27 (**Table 1**). Leaf potassium concentration was reduced by salinity at both T27 and T37 but not at T42 (**Table 1**). Under 100 mM, T42 plants displayed the highest leaf potassium concentration while, under 0 mM NaCl, leaf potassium concentration was the highest in the T37 treatment (**Table 1**). Leaf calcium concentration was only affected by T42 with a significant increase when compared to T27 but was not changed by salinity (**Table 1**). The treatments and their interaction did not affect leaf magnesium concentration (**Table 1**).

**Table 1:** Analysis of variance and mean comparisons for leaf nutrient concentrations of the African eggplants grown under different salinity and temperature levels.

Source of variance	Na	N	P	K	Ca	Mg	Mn	Cu	Zn	Fe
	g kg^-1^						mg kg^-1^			
Salinity (S)	***	**	ns	***	ns	ns	ns	**	ns	***
Temperature (T)	***	***	***	ns	**	ns	***	**	**	***
S x T	ns	ns	ns	*	ns	ns	**	ns	ns	*
Salinity										
0 mM NaCl	0.27^b^	60.20^a^	7.55	43.70	25.77	6.43	110.1	2.26^b^	25.45	96.52
100 mM NaCl	12.27^a^	55.08^b^	7.43	31.70	29.40	7.17	108.3	3.01^a^	30.02	82.35
Temperature										
T27	2.45^b^	63.14^a^	6.67^b^	36.14	23.20^b^	7.00	68.08	2.04^b^	22.48^b^	98.47
T37	7.31^a^	57.72^b^	8.03^a^	39.42	25.24^ab^	6.54	123.1	3.18^a^	34.20^a^	103.9
T42	9.04^a^	51.81^c^	7.78^a^	37.55	34.31^a^	6.87	136.4	2.69^ab^	26.53^b^	65.91
S x T										
0 mM + T27	0.045	66.94	6.44	41.75^ab^	18.89	6.01	58.24^b^	1.63	20.75	101.7^ab^
0 mM + T37	0.23	60.80	8.26	48.39^a^	25.27	6.68	123.2^a^	2.76	31.35	117.9^a^
0 mM + T42	0.55	54.84	7.94	40.94^bc^	33.14	6.60	148.8^a^	2.40	24.26	69.91^c^
100 mM + T27	4.86	60.28	6.89	30.52^d^	27.51	7.98	77.92^b^	2.45	24.22	95.24^b^
100 mM + T37	14.40	56.18	7.79	30.44^d^	25.22	6.40	122.9^a^	3.61	37.05	89.91^b^
100 mM + T42	17.54	48.78	7.62	34.16^cd^	35.48	7.13	124.1^a^	2.99	28.79	61.90^c^

Na, Sodium; N, Nitrogen; P, Phosphorus; K, Potassium; Ca, Calcium; Mg, Magnesium; Mn, Manganese; Cu, Copper; Fe, Iron; Zn, Zinc. NS, *, **, *** Non-significant or significant at P ≤ 0.05, 0.01, 0.001, respectively. Means not sharing any letters within each column are statistically different according to Tukey's test (P = 0.05).

Leaf manganese concentration was increased by T37 and T42 under both salinity levels (**Table 1**). Leaf copper concentration was increased by salinity and by T37 significantly but not by T42 when compared to T27 (**Table 1**). T37 was also the only treatment to increase leaf zinc concentration while T42 and salinity did not impact it significantly (**Table 1**). Under 0 mM NaCl, leaf iron concentration was initially increased by T37 from T27 but decreased significantly at T42 (**Table 1**). The initial increase was not observed under 100 mM NaCl but the decrease at T42 was also present (**Table 1**). Salinity also decreased leaf iron concentration for T37 plants (**Table 1**).

## 4 Discussion

The harmful or beneficial effects of salinity and heat have been reported for a range of crops but their combination is still only sparsely studied (Suzuki et al., 2014). In this study, while some plant parameters were affected by the individual stresses and not further impacted by the stress combination, such as chlorophylls and shoot dry weight, other traits responded differently to the combination of heat and salinity, in particular leaf fluorescence parameters.

Despite a slight decline in leaf expansion rate, salinity did not impact the final leaf production in the current study, which contrasts with what was previously observed in other *Solanum* species (Efimova et al., 2018; Alsafari et al., 2019; Ortega-Albero et al., 2023). Bacha et al. (2017) observed a decrease in leaf area after 14 days of stress while no differences were noted after 7 days, highlighting the role of salinity build-up and tolerance variation through time. In the current study, both high temperatures reduced leaf dry weight but leaf expansion rate and leaf number were only reduced at 42°C, suggesting another reason involved in the reduction observed at 37°C. A similar pattern was noted in a tomato (*S. lycopersicum* L.) cultivar with no reduction in leaf area and leaf number but a reduction in final shoot dry weight under heat (Zhou et al., 2017). The production of thinner leaves at high temperatures to reduce leaf temperature, despite its impact on plant water status and membrane stability, might explain the reduction in leaf dry weight (Schiattone et al., 2017).

Salinity decreased membrane stability in the current study as in Ben Abdallah et al. (2016) study on *S. nigrum* L. Shunkao et al. (2022) reported a further decrease in membrane stability when both heat and salinity when present in wheat (*Triticum aestivum* L.), similar to what was observed in this current study at T42. However, T37 had a mitigating effect with a non-significant decrease in membrane stability which might be due to a level of protection offered by a moderate increase in temperature. Despite studies having previously shown that heat can have a protective effect against salinity on photosynthesis or sodium transport rate, similar observations in membrane stability have yet to be made (Rivero et al., 2014; Lopez-Delacalle et al., 2021). The protection effect observed in this current study might be linked to the increased leaf zinc concentration under moderate heat, a mineral element previously linked to improving membrane stability under stress (Tufail et al., 2018; Hassan et al., 2020). In accordance with the protective effects of temperature on membrane stability only appearing at T37, the increased leaf zinc concentration was not noted at T42, at which point membrane stability was reduced. Surprisingly, temperature alone did not impact membrane stability despite the negative effects of T42 on other measured characteristics being significant. Electrolyte leakage is positively correlated to the reactive oxygen species accumulation and lipid peroxidation, not measured in the current study, and its maintenance is generally a sign of tolerance (Demidchik et al., 2014). The negative effects of T42 observed in this current study on parameters depending on membrane stability such as chlorophylls are, however, a sign of its irrelevance regarding the overall tolerance of the African eggplant. These negative impacts might be due to other damaging processes under heat such as enzyme inhibition or transcriptional changes (Zhao et al., 2020). It has to be noted, however, that midday stomatal conductance was maintained, and even increased, under the T42 treatment. As F1 Djamba is marketed as a particularly high-yielding cultivar, mechanisms might be in place to maintain gas exchange and carbon assimilation even under stress, thus requiring sustained membrane stability. The maintenance of membrane stability might thus have been important for characteristics not measured in the current study and require further research.

Midday stomatal conductance was increased by the T42 treatment seven days after the beginning of the stress but not by the T37 treatment, suggesting different mechanisms in place to cope with high temperatures depending on their intensities. The increase in stomatal conductance, despite increasing water loss, helps regulate leaf temperature to maintain the activity of photosynthetic enzymes (Poudyal et al., 2018). This increase was noted in different tomato cultivars at lower temperatures, highlighting the high tolerance threshold of the African eggplant (Poudyal et al., 2018; Amuji et al., 2020). The increased stomatal conductance promoted by high temperatures was, however, reduced under salinity. This was previously observed in tomato plants where the lowest stomatal conductance was reached under the stress combination due to the osmotic stress created by the salinity stress, leading to mechanisms in place to limit water loss such as stomatal closure (García-Martí et al., 2019).

Plants grown under higher temperatures kept their stomata closed for longer at the start of the day in this current study. Similarly, the largest difference between drought-stressed and non-stressed *Carapa guianensis* Aubl. was observed early in the day (Carvalho et al., 2013). Stomatal conductance of sorghum (*Sorghum bicolor* L.) was reduced early as well but did not increase back to control levels during the day, unlike the observations made in the current study (Sonobe et al., 2009). The current observations may be due to adaptive mechanisms under which the plants only fully open their stomata after reaching a high level of light to balance water loss with carbon gain. However, it has to be noted that the temperature started to reduce eight hours after the start of the photoperiod as per the gradual increase and decrease temperature set-up. While still the highest temperature, T42 treatment was at 40°C at that time, reduced from 42°C which only lasted from the 2^nd^ to the 6^th^ hour after the start of the photoperiod. A reduction in temperature is linked to a reduction in vapor pressure deficit (VPD), allowing better gas exchange (Merilo et al., 2018). In this current study, the 2°C reduction from 42°C might be enough for the plants to recover stomatal conductance. Even if the VPD of the other treatments also decreased at this time, the change might not have improved stomatal conductance as the VPD was already within optimal levels, leading to T42 plants recovering to the levels of the other treatments.

In addition to stomatal conductance changes, the stresses in the current study impacted the photosynthesis apparatus through chlorophylls and fluorescence modifications. While SPAD and final chlorophylls were significantly reduced by T42, leaf fluorescence parameters were not drastically reduced by this treatment. This observation might be due to the relationship between chlorophyll content per area and leaf thickness (Jinwen et al., 2009). Leaf thickness is positively correlated with SPAD index but negatively correlated with *ϕ*2 due to loss of transmittance at high leaf thickness which limits the optimal utilization of each chlorophyll molecule (Knapp and Carter, 1998; Jinwen et al., 2009). In the current study, plants with a lower SPAD (under the T42 treatment) may have a higher light use efficiency, leading to the mitigated impact of the stresses on *ϕ*2 and *ϕ*NPQ despite the noticeable loss of chlorophyll molecules.

In terms of biochemical changes, the increase in total carbohydrates under heat was also observed by Zhou et al. (2017) in tomato. In contrast, no changes in glucose and fructose were noted under heat by Botella et al. (2021) in tomato but salinity increased these compounds significantly, while the stress combination further increased them. The increase noted by other studies was attributed to the enhanced enzyme activity within the sugar biosynthesis pathways, the insufficient sink activity limiting the export of the produced sugars from leaves, and the role of sugars as osmoprotectants (Zhou et al., 2017; Botella et al., 2021). In this current study, heat might have activated one or a combination of the stated accumulating mechanisms to withstand stress but not salinity. Salinity also did not trigger a non-enzymatic antioxidative response with the absence of changes in antioxidants and a reduction in total phenols. Phenols and antioxidants are important to detoxify reactive oxygen species created under stress and are sometimes a key part of the tolerance mechanisms (Das and Roychoudhury, 2014). The observations made in this current study were in line with the results of Martínez et al. (2020) who reported no changes in antioxidants in tomato and *S. chilense* under salinity. Phenols were increased by salinity in tomato (Bacha et al., 2017) but not in brinjal eggplant (*S. melongena* L.) and only at 50 mM NaCl in *S. nigrum* (Ben Abdallah et al., 2016; Ortega-Albero et al., 2023), showing the importance of the cultivar of interest and the level of stress.

Sodium accumulation under salinity, a quick and common observation, has major negative impacts on plant development (Sousa et al., 2022). In the current study, leaf sodium concentration was highly increased under salinity, an increase further exacerbated under heat as seen previously in tomato plants (Sousa et al., 2022). The antagonist effect of plant’s sodium concentration on calcium uptake was not seen in this current study despite previous reports of calcium reduction in *Solanum* under salinity (Ben Abdallah et al., 2016; Ben-Abdallah et al., 2019; Sousa et al., 2022). The decrease in leaf calcium concentration in *S. villosum* Mill. reported by Ben-Abdallah et al. (2019) was, however, only seen under 150 mM NaCl and not at 50 or 100 mM NaCl, suggesting a threshold under which its uptake is not hindered in leaves. Leaf potassium and phosphorus concentrations were reduced under heat in the study by Ali et al. (2021), which was not observed in the current study with leaf phosphorus and potassium concentrations increased and unchanged, respectively. This corroborates findings in the brinjal eggplant and potato (*S. tuberosum* L.) (Efimova et al., 2018; Ortega-Albero et al., 2023). The maintenance of these primary nutrients under stress is important for enzyme activity, cell membrane stability, and reactive oxygen species detoxification among others and the variability of responses under stress highlights the difference in mechanisms triggered for each stress (Kumari et al., 2022). Leaf nitrogen concentration, another primary nutrient, was reduced under salinity only at T27 and T42 but not at T37. Nitrogen has key roles in various tolerance plant mechanisms and moderate temperature stress might improve its uptake or transport to enhance tolerance through improved membrane stability or photosynthetic activity (Kumari et al., 2022).

Despite the role of magnesium in enzyme activity maintenance, the abiotic stresses did not affect leaf magnesium concentration in this study as was reported previously, suggesting its non-primary role in *Solanum* stress response (Ben-Abdallah et al., 2019). Leaf zinc and copper concentrations were only increased at T37 when compared to T27, while T42 levels were similar to the T27 ones. The increase seen only at T37 might be due to the increased nutrient uptake of selected important nutrients for tolerance observed under a certain threshold in different species due to the maintained ATPase activity and increased water uptake to compensate for the higher transpiration, mechanisms potentially not in place at T42 in the current study (Klock et al., 1996; Dias and Lidon, 2009). Interestingly, the temperature experienced by the roots was not significantly different between the T37 and T42 treatments, both much higher than the T27 treatment (data not shown). The reduction in leaf zinc and copper at T42 when compared to T37 was thus not due to an increase in heat damage on the roots which commonly reduces nutrient uptake (Bravo-F and Uribe, 1981; Mishra et al., 2023). This observation might instead be due to a translocation mechanism not impacted at 37°C but hindered at 42°C. Indeed, the translocation of zinc and copper from roots to shoot and the different plant organs relies on a range of proteins which might be denatured under high temperatures (Gupta et al., 2016; Mishra et al., 2023). The promotion of nutrient uptake alongside denatured proteins might thus have led to the same leaf zinc concentration observed between T27 and T42, both lowered that in T37 where the nutrient uptake was increased without damaging effects of protein denaturation. It has to be noted that copper, zinc, and manganese play an important role in the activity of the antioxidant enzyme superoxide dismutase (Tyagi et al., 2019). The increase observed at T37 might be part of tolerance mechanisms increasing the concentrations of important nutrients to detoxify reactive oxygen species accumulated during the stress with the increase in non-photochemical quenching and other damage. Further investigation is required to better understand the role of zinc, copper and manganese in the African eggplant under heat stress and their potential impact on antioxidant enzymes.

## 5 Conclusion

The current study aimed to describe the effects of the stress combination on an African eggplant Kumba cultivar and showed that despite some unique responses under the combination of heat and salinity, several plant attributes followed the same trend as the individual stresses. Elevated temperatures impacted highly leaf weight, polyphenols, and chlorophyll, salinity mostly had an effect on electrolyte leakage, stomatal conductance, and sodium uptake, while the stress combination impacted *ϕ*2, *ϕ*NPQ, and potassium. The level of heat stress was important in dictating whether the stress combination had a stronger negative effect than the individual stresses, highlighting the importance of stress intensity when multiple stresses are present. Moderate heat offered some protection against salinity stress on the African eggplant regarding cell membrane stability, leaf fluorescence, and zinc and potassium levels, highlighting some positive effects of stress combination under certain conditions. The African eggplant did not rely on non-enzymatic antioxidant mechanisms to maintain photosynthetic activity, leaf expansion or chlorophyll levels, suggesting other mechanisms triggered under stress to maintain these processes.

The current study showed that F1 Djamba supported well a 37°C air temperature on its vegetative development but not 42°C. This is an important observation for farmers to adapt their planting habits following heat wave predictions. In addition, this is relevant to breeders aiming at developing tolerant crops as some tolerance pathways have been highlighted such as polyphenols and chlorophyll adaptation. The predominance of either salinity or heat on certain characteristics is also important for breeders and researchers to understand whether a crop tolerant to one stress can be tolerant to a combination of stresses.

## Data availability statement

The original contributions presented in the study are included in the article/supplementary material. Further inquiries can be directed to the corresponding author.

## Author contributions

ND-R: Conceptualization, Investigation, Methodology, Writing – original draft. MB: Supervision, Validation, Writing – review & editing. ES: Supervision, Validation, Writing – review & editing.
